# The effects of low-dose radiation on articular cartilage: a review

**DOI:** 10.1186/s13036-018-0125-4

**Published:** 2019-01-07

**Authors:** Hannah Cash, Delphine Dean

**Affiliations:** Department of Bioengineering, Clemson University, 301 Rhodes Research Center, Clemson, SC 29634 USA

**Keywords:** Orthopedics, Cartilage, Radiation, X-rays, Gamma rays, Ionizing radiation

## Abstract

Articular cartilage is a specialized connective tissue, predominately composed of water, collagen, and proteoglycans, that provides a smooth, lubricated surface for articulation in joints. It has long been considered radioinsensitive and therefore unaffected by exposure to radiation in medical settings. Due to the increased amount of yearly radiation exposure through radiotherapy and ionizing radiation diagnostic procedures, there has been a renewed interest in how radioinsensitive articular cartilage actually is. Despite this renewed interest, the majority of these studies do not focus on articular cartilage as their primary goal, but rather, have observed the effects of total body irradiation. Since many of these studies do not report the type of irradiation used, the rate of exposure, or use consistent models, there are inconsistencies in these studies, which make comparing and translating the results difficult. Previous literature reviews have found less than 60 studies discussing the effects of radiation on articular cartilage and its components both in vitro and in vivo*.* However, despite the inconsistencies, these reviews and studies have drawn the same overall conclusion that this research needs to be continued and broadened in order to make a consistent conclusion on the radioinsensitivity of articular cartilage. Therefore, the goal of this review is to categorize and summarize current findings in literature discussing the effects of radiation on articular cartilage.

## Introduction

The amount of radiation that a person is exposed to yearly has dramatically increased over the last few decades [1]. In the United States, the average yearly medical exposure dose increased from 0 to 5 mGy in 1982 to 30 mGy in 2006 and is expected to continue increasing [1]. In the United Kingdom, the average yearly medical exposure dose doubled from 1982 to 2006 and tripled in Australia from 1982 to 2006 [1]. Therefore, understanding the effects of radiation on tissue is vital for protecting individuals exposed to radiation.

There are two types of radiation: nonionizing and ionizing. Ionizing radiation is considered more dangerous because it produces charged particles called ions that can cause cells to prematurely die or mutate incorrectly and become cancerous [1]. Individuals are constantly exposed to ionizing radiation due to its use in the medical field for imaging, radiotherapy, and sterilization [2–4]. In this review, we will discuss recent findings in the literature discussing the effects of ionizing radiation on articular cartilage.

Articular cartilage has previously been considered radioinsensitive and therefore considered safe when exposed to radiation in medical settings [5–8]. Articular cartilage is a specialized connective tissue composed of hyaline cartilage that provides a smooth, lubricated surface for articulation in joints [9]. It is predominately composed of water, collagen, and proteoglycans [9]. The unique balance between these components is essential to keep the biomechanical properties of the tissue intact since articular cartilage does not have a direct supply to blood vessels, lymphatic drainage, or a neural connection linked to the homeostatic system [10]. Thus, injury response of the tissue is ineffective.

Currently, a number of studies have shown that radiation does not have an adverse effect on articular cartilage, but these studies have mainly studied the apoptotic effect of ionizing radiation [11, 12] (see Table 1). The apoptotic effect of radiation is the main focus of numerous studies since a major goal of radiotherapy is to enhance the efficacy of ionizing radiation in tumors [13]. One study has showed that 2 cGy does not induce cell death and that low-dose radiation does not have “pathological effects on primary cultured articular chondrocytes” as well as that low-dose radiation may be a beneficial therapeutic option for cartilage diseases [2]. Another study found that apoptosis was induced after ionizing radiation exposure in degenerated cartilage, but not in nondegenerated cartilage [14]. A similar study also found that apoptosis was not induced in articular cartilage even when it was exposed to 10Gy gamma radiation [15]. These findings were confirmed by Moussavie-Harami et al., Kim et al., Takahashi et al., and Ogawa et al. who found that apoptosis was not induced in articular cartilage that was exposed to gamma irradiation doses of 5Gy, 10Gy, and an X-ray irradiation dose up to 30Gy respectively [16–19].

**Table 1:** The effects of the four types of low-dose radiation used in clinical settings on articular cartilage characterized at positive, negative, or no effects

Type of Ionizing Radiation	Effects of Low-Dose Radiation on Articular Cartilage		
	Positive	Negative	None
Beta		✓ [41, 48]	
Gamma	✓ [11, 16, 27, 51]	✓ [13, 26, 46–48, 52]	
X-Ray	✓ [38]	✓ [15, 20, 27, 43, 44]	✓ [36, 51]

However, despite these studies, there are studies that show that low-dose radiation does affect articular cartilage [20–25]. In another study performed by Hong et al., researchers found that when articular cartilage was exposed to gamma radiation at a dose rate of 3.81 Gy/min, cellular senescence was induced. Two studies found that when articular cartilage is exposed to a dose of 2Gy gamma radiation, active degradation of the cartilage occurs [20, 26]. Willey et al. found that irradiation lowered the synthesis of proteoglycans, induced active degradation of the matrix, as well as arthropathy and Lindburg et al. found similar results in that low-dose radiation caused changes in the mechanical properties of articular cartilage, which may be due to the acute release of glycosaminoglycans [20]. Another similar study also found that when articular cartilage was exposed to 3 and 7Gy, degenerative changes to the tissue were observed [27].

As seen in Tables 1 and 2, the four types of ionizing radiation used in clinical settings have been found to have a wide range of effects on articular cartilage, ranging from no effects to positive effects to negative effects. However, the majority of these studies are not focusing on articular cartilage as their primary goal, but rather, are observing the effects of total body irradiation. Due to the majority of these studies not consistently reporting the details of their experiments, there are inconsistencies between studies, which makes comparing and translating the results challenging. Previous literature reviews have found less than 60 studies discussing the effects of radiation on articular cartilage and its components and our current search found less than 75 journal publications with similar search criteria [8]. While most studies report the total dose administered, there is sometime no other detailed information on the radiation used in these studies (e.g., type, power, dose rate). Yet, despite the differences in the literature, authors have drawn the same overall conclusion that more research needs to be continued and broadened in order to make a consistent conclusion on the effects of radiation of articular cartilage [8]. Thus, there is currently a large gap in literature that needs to be bridged in order to better understand the effects of radiation on articular cartilage.

**Table 2:** Common effects of radiation on articular cartilage

Type of Ionizing Radiation	Effects
Beta	Cell viability [48]
Gamma	Cell death [13], cell cycle arrest [11], anti-inflammatory [37, 43], matrix degradation [5, 26, 27]
X-Ray	Anti-inflammatory [38], chondrocyte proliferation [15], anti-inflammatory [38]

### Common reported radiation doses

The type of radiation and dose vary widely depending on the exposure route (Table 3). It should be noted that radiation exposure is often reported in Sv, as opposed to Gy. Grays represent the absorbed dose. Sieverts represent the equivalent biological dose. For photon radiation (x-ray and gamma) and beta particles, Sieverts and Grays are equivalent as the radiation weighting factor for these is 1. In contrast, heavy nuclei and alpha particles have weighting factor of 20 meaning that the equivalent dose in Sv is 20 times the absorbed dose in Gy. This weighting factor takes into account the varying biological effects of different radiation types.

**Table 3:** Radiation exposures and doses found in the literature

Exposure	Typical Total Dose	Type of Radiation
Hand, foot, dental x-ray imaging	0.001–0.01 mSv	x-ray [53–56]
Mammography	0.4 mSv	x-ray [53–56]
Computed Tomography (CT)	2–30 mSv	x-ray [53–56]
6-month stay on ISS station	80 mSv	Solar particles, cosmic rays [53–56]
6-month trip to Mars	250 mSv	cosmic rays [53–56]
Highest dose received by Fukushima emergency worker	670 mSv	alpha, beta, and gamma [56]
Fractionated radiotherapy dose	1-2Sv	x-ray or gamma [56]
Total radiotherapy dose	60 Gy	x-ray or gamma [56]
Radiosurgery	60–80 Gy	x-ray or gamma [56]
Radiation for sterilization	25 kGy	Gamma [31]

## Extremely high-doses of radiation

Two studies have examined the effects of an atomic bomb, which produces neutron radiation. The first study examined delayed effects of atomic bomb radiation in mice [28]. The radiation was measured to be ~ 0.250Gy of neutrons at ~ 1 km away from the epicenter. Animals closer to the blast did not survive more than 24 h. The study concluded that instantaneous exposure to an atomic bomb sets into motion changes that culminate into the premature onset of natural senescence diseases as well as shortens the life span of the mice [28]. These diseases included cartilaginous diseases, such as osteoma, osteogenic sarcoma, and osteosarcoma [28]. The second study discussed the long-term health effects of radiation in the human population affected by the atomic bomb [29]. This study drew similar conclusions as the mice atomic bomb study. The two main conclusions drawn were that cancer risk increases in those who were younger when exposed to the atomic bomb and that continued research needs to be conducted in order to determine the extent of the health effects [29].

Another study conducted at extremely high doses, discussed the effects of gamma radiation on human costal cartilage and its effects on the biomechanical properties. Their study was one of the only studies to report the type of source used, a cobalt source [30]. They found that at 15 kGy, there was no significant effect on the biomechanical properties of the costal cartilage, but doses between 40 kGy and 50 kGy may affect the biomechanical properties [30].

In addition, there have been some studies on the effect of the gamma radiation used in sterilization on the mechanical properties of orthopedic tissues [31]. These high doses of gamma are used to kill cells and microorganisms for allografts and biomaterials used in implants [31]. Studies have found that dose higher than 25 kGy also change the mechanical properties of bone, tendon, and ligament by altering the molecular structure of the tissue matrix [31].

## High-dose radiation

### No effects

The majority of high-dose radiation studies conclude that high-doses of radiation cause significantly negative effects on tissue, including articular cartilage. However, two studies concluded that high-dose radiation causes no effect on articular cartilage. The first study irradiated the tibia of chick embryos at 20Gy increments up to 200Gy [32]. This study did not report the type of ionizing radiation used. It was concluded that up to a 150Gy dose, secretion of new proteoglycans was not affected [32]. The second study examined long-lasting tolerance of articular cartilage in the knee joints of adult rabbits. This study used a single dose of 50Gy X-ray for each exposure [33]. The study observed the return of normal cartilage architecture 15 months post-irradiation [33]. It was then concluded that articular cartilage tolerated intraoperative radiotherapy without sustaining any serious degenerative changes [33].

### Negative effects

The negative effects of ionizing radiation have been well documented. However, the specific effects of ionizing radiation on articular cartilage have not been studied as in depth as the overall effects of ionizing radiation.

### Unreported type of ionizing radiation

Various other studies have examined the overall effects of high-doses of radiation, but few have specifically examined the effects on articular cartilage. Of those studies, the majority do not report the type of ionizing radiation used or the dosage used in the experiment.

For instance, researchers looked at the effects of radiation on the matrix synthesis in non-ossifying chick embryonic cartilage [22]. This study did not report the dosage or the type of ionizing radiation used [22]. The study concluded that there was an increase in proteoglycan synthesis and that there was dose- and time-dependent necrosis observed [22]. Another study looked at the effects of ionizing radiation and hyperbaric oxygenation on rabbit mandibular condylar cartilage. This study did report that the dosages used were a low-dose of 2.2Gy and a high-dose of 50Gy in 25 fractions [34]. However, this study did not report the type of ionizing radiation used. The study concluded that the cartilage was partially or totally devoid of proteoglycans and that the architecture of the cartilage was severely damaged after radiation exposure [34].

### Gamma radiation

The high dose studies that did report the type of ionizing radiation used, mainly used gamma radiation. However, these studies did not use the same model, the same type of radiation source, or the same dose rate. Schönmeyr and colleagues discussed the effects of gamma radiation on mesenchymal stem cells. They found that the majority of mesenchymal stem cells survived, but went into G2 cell-cycle arrest and became senescent or terminally differentiated toward the bone lineage [35]. Overall, the study concluded that there was a decrease in cellular proliferation and that the cells became resistant in cellular survival while their function was markedly altered [35].

## Low-dose radiation

There has been a recent increase in the number of studies examining the effects of low-dose radiation on articular cartilage. However, there is an inconsistency in the type of ionizing radiation, the dosage, the dose rate, the source, and the model used. Therefore, the conclusions of each study are difficult to translate and thus draw an overall conclusion about the effects of low-dose ionizing radiation on articular cartilage. Due to the inconsistencies between studies, the results are vastly different with various studies concluding no effects on articular cartilage, others reporting positive effects, and others reporting negative effects.

### No effects

One study observed the effects of low-dose radiation on the structural and mechanical properties of hyaline cartilage-like fibrocartilage in mature female rabbits. The study used 1Gy fractions per day for 5 days using 6MV photon (x-ray) radiation [36]. It was concluded that radiotherapy applications to hyaline cartilage-like fibrocartilage tissue did not change its mechanical properties in vivo [36]. However, the study made an overall conclusion that more comprehensive studies with a longer follow-up and larger sample size needs to be conducted [36].

### Positive effects

Other studies have concluded that low-dose radiation may have positive effects on articular cartilage. Hong et al. studied the effects of low-dose gamma radiation on rats. The dosage and source used in this study was not reported. They observed that the effects of the ionizing radiation might have a helpful effect on the modulation of DNA damage, longevity, and immunological responses [11]. The study also reported improvements in the joint swelling and pain of the mice [11]. However, despite stating that the low-dose gamma radiation may have had positive effects, the study makes an overall conclusion that no clear conclusion can be drawn because the molecular mechanism underlying the observed anti-inflammatory effects was not understood [11].

Despite the previous study’s general conclusion that no overall conclusion could be drawn one study has observed positive effects of low-dose radiation on cartilage [37]. In a 2009 review article, Richardson examined the effects of various types of ionizing radiation on various animals. The review found that there have been contradictory results in animal and human studies [37]. The studies that the review discussed primarily used mice models and doses between 1 and 20Gy [37]. Most of the studies did not focus on cartilage. The review concluded that low-dose radiation demonstrated hormesis health benefits in some cases, but further research needs to be conducted to fully understand the health effects [37].

Steffen et al. demonstrated positive effects of low-dose radiation in a rabbit study that examined the influence of X-ray treatment on antigen-induced experimental arthritis [38]. The right knee of tree groups of rabbits was exposed 12 days after intra-articular challenge to 6Gy for 8 min, the equivalent of a dose rate of 750 mGy/min [38]. The study reported that the irradiated rabbits showed little to no synovitis 48 h and 7 days following exposure whereas nonirradiated animals showed distinct chronic synovitis [38]. The study recommended that X-ray irradiation treatment for inflammation in arthritic joints should be reconsidered [38].

Another study examined the effects of 0, 1, 2, 3, and 5Gy gamma exposures using a 10,000-Ci ^137^Cs irradiator at a dose rate of 1Gy/minute in human chondrosarcoma cells [16]. The study found that the chondrosarcoma cells were resistant to clinically applicable doses of gamma irradiation [16]. The study attributed this to the absence of effective p16 tumor suppressor activity [16]. The study also found that there was an increased sensitivity to radiation, which may lead to an increased effectiveness in radiation treatments for patients with chondrosarcomas [16].

There are few long term studies in humans with low dose radiation. Keller et al. examined the efficacy of low-dose radiotherapy in painful gonarthritis through a retrospective study in East Germany. The clinical data consisted of 1037 patients who underwent radiotherapy with orthovoltage units, a linear accelerator, or a Cs-137 radiation source in the 1980s [39]. The regiment consisted of either once a week in series, twice a week in series, or daily with single doses ranging between 0.5Gy to 1.5Gy for one series [39]. The study concluded that their results confirmed that low-dose radiotherapy was effective at treating painful osteoarthritis in the knee, but that the influence of radiobiological severity for treatment outcomes remained unclear [39]. When comparing the study analysis to previous retrospective analysis, the results were contradictory [39]. Therefore, the study concluded that more research needs to be conducted to determine the efficacy of low-dose radiotherapy in treating osteoarthritis in the knee [39].

#### Negative effects

Even though some of the studies mentioned above [11, 16, 37–40] have shown either no effect or some positive effects of ionizing radiation on articular cartilage and cells, more recent studies have shown that ionizing radiation can have negative effects articular cartilage. However, there is a lack of consistency between the type of radiation, the dosage of radiation, the dose rate, and the model used.

#### X-ray radiation

Numerous studies have chosen to study the effects of X-ray radiation due to their predominate use in clinical settings. Despite numerous studies using X-ray radiation, there still is not a consistent dose, dose rate, energy/wavelength, or model used throughout the studies.

**Fig. 1:**
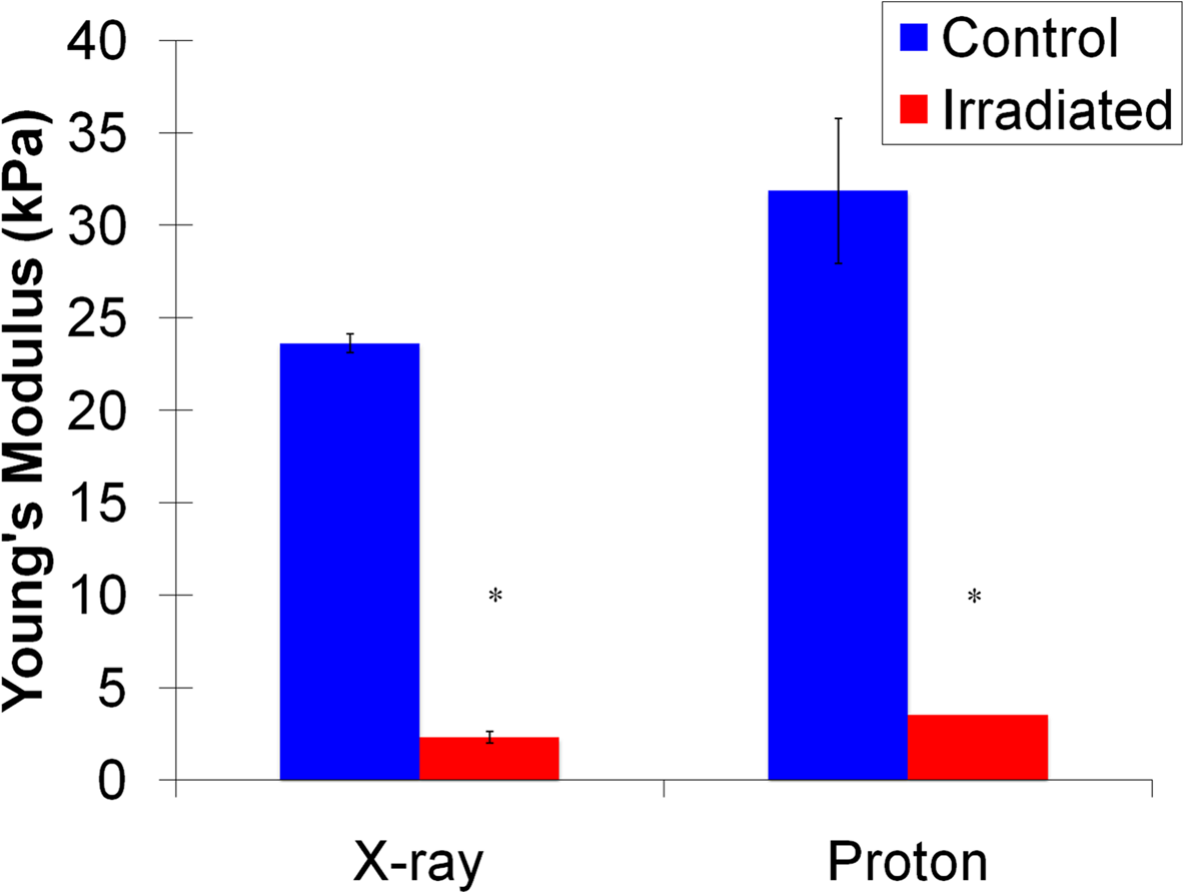
The Young’s Modulus of the control (blue) and irradiated (red) cartilage (adapted from Lindburg 2012) [22]. The modulus was calculated from the atomic force microscopy indentation of cartilage from mice 7 days after 2Gy of X-ray exposure or 2 days after 3Gy of proton exposure. The Young’s Moduli for the irradiated samples were significantly lower the non-irradiated controls

Lindburg et al. examined the effects of low-doses of X-ray radiation exposure on the metabolic and mechanical properties of mice and porcine articular cartilage using a dosage of 2Gy [20]. It was found, as seen in Fig. 1, that this dosage of ionizing radiation caused adverse effects on the functional properties in both the mice and porcine models [20]. However, an overall conclusion was stated that further research must be performed to determine the mechanisms of the damage [20].

Another study used varying doses of 1Gy, 3Gy, and 7Gy X-rays on total-body irradiation of 14-week-old rats. This study examined late degenerative changes in articular cartilage and in bone [27]. The study identified potential prognostic indicators of late radiation-induced joint damage in the mice [27].

Another popular model used in radiation studies is the rat model. Melanotte et al. examined the early effects of X-ray exposure on the bone and cartilage of 60 albino rats. The dose rate used was 1.209Gy/minute using a Westinghouse constant potential dual X-ray machine [41]. The study concluded that there was prompt damage to cartilage by the cessation of growth and alterations in some of the histochemical reactions [41]. Willey et al. used 16 weeks old male Sprague Dawley ® rats to examine the effects of a 1Gy total-body exposure of X-rays in combination of limb unloading in order to simulate spaceflight [42]. The study found that there was acute degradation of cartilage in the knees and that recovery was limited after an extended period of reloading [42]. The study concluded that pre-arthritic changes might lead to the development of symptomatic arthritis [42].

Numerous X-ray studies use chondrocyte cell culture models to determine the effects of X-ray exposures. Matsumoto et al. discussed the effects of a single dose of a 2Gy and 10Gy X-ray exposure on 21-day-old rabbit chondrocytes [43]. They concluded that the synthesis of cartilage matrix components, predominately the proteoglycans, was relatively radioresistant in comparison to the synthesis of DNA [43]. However, they observed that the radiation exposures affected the proliferation and differentiation of the chondrocytes [43]. Hamdi et al. examined the effects of a 2Gy/minute dose rate of X-ray exposure and a 1Gy/minute dose rate of LET of carbon ions on human articular chondrocytes. The study induced a comparable rate of senescence in the three-day model, which suggested that carbon ions could successfully treat tumors that are resistant to traditional radiation therapy [44]. However, the primary conclusion drawn was that the scientific community needs to use relevant models in order to determine better safety measures for patients [44]. In contrast, Margulies et al. examined the effects of radiation therapy on primary rat costochondral growth cartilage chondrocytes. This study used a dose rate of 2.09 Gy/minute with a 300 kV and 10 mA X-ray source with doses of 0, 1, 2, 5, 10, and 20Gy [15]. The study focused on the effects of the radiation on the proliferative chondrocytes and found that the irradiation exposure may negatively affect the pathway that regulates chondrocyte sensitivity to hypoxia [15]. The authors made an overall conclusion that more work needs to be performed to determine the effects of irradiation [15].

#### Beta radiation

Very few studies have focused on the effect of beta irradiation on cartilage. Beta irradiation is commonly used clinically for brachytherapy as well as in certain industrial processes such as plastic films [45]. One study that used beta irradiation, studied incubating bovine synovial membrane from elbow joints with 0 to 3 MBq ^90^Y/ml medium [40]. The study’s main conclusion was that the long-term effect of cell viability may be influenced by beta irradiation, but the overall conclusion was that further studies need to be conducted to determine if low-dose radiation affects the survival of cells and their potential to recover and return to normal cellular activities [40].

#### Gamma radiation

It is important to determine the effects of gamma radiation on articular cartilage due to the prevalence of gamma radiation in radiotherapy and in certain imaging modalities. Those studies that do use gamma radiation predominately reported the type of irradiator, the dose, the dose rate, and the model used. Reporting this information is essential in effectively translating the conclusions drawn in each study in order to determine an overall conclusion on the effects of low-dose gamma radiation on articular cartilage.

The two most common models used in these gamma studies were rabbit and human models. A recent study by Gönç et al. examined the effects of radiation on New Zealand White forty-month-old female rabbit osteochondral allografts [46]. The study dosed each rabbit five times with a dose of 100 cGy using a Co-60 gamma irradiator [46]. This study found that this fractionated dose resulted in less chondrocyte damage, but there were adverse effects on the incorporation of the graft to the host [46]. Overall, the study concluded that the optimal dose regimen for immune suppression through fractionated radiotherapy should be further studied [46]. Hong et al. examined the effects of a dose rate of 3.81 Gy/minute using a Cs-ray source on primary rabbit articular chondrocytes [13]. This study concluded that this dose rate induced cellular senescence of the articular chondrocytes and that further studies are needed to identify the molecular mechanisms that are causing the induction of cellular senescence [13].

There were two types of human models used, chondrocytes explanted from ankles and a comparison of healthy and cancerous human articular cartilage. Willey et al. examined the effects of doses of 2Gy and 10Gy delivered by a Cs gamma irradiator at a dose rate of 3.64Gy/minute on human ankle chondrocytes [26]. They found that there was a decreased amount of proteoglycans synthesized as well as an induction of matrix degradation [26]. The study made an overall conclusion that the low-doses of radiation may cause functional decline of cartilage health, however, more studies need to be conducted to confirm this conclusion [26]. Kyriakidou et al. utilizing a human cartilage model, examined the effects of a 2Gy dose up to a 60Gy dose of radiation delivered with a Cobalt gamma irradiator to healthy and to cancerous human articular cartilage. This study found that the radiation changed the length of the proteoglycans by changing the length of the sugar chains [47]. The study also found that the proteins in the cartilage changed from an alpha helix arrangement to a random coil and then to an amyloid-like protein, which ultimately lead to fiber formation [47].

## Discussion

The amount of medical radiation that an individual is exposed to has increased at a rate of 20.3% mGy per year over a 24-year period [48]. Due to this dramatic increase, it is essential to understand the effects of radiation on tissues that have been previously thought to be radioinsensitive, such as bone and articular cartilage [37]. Thus, there has been an increase in the number of studies examining the effects of radiation on articular cartilage. However, there a number of inconsistencies in these studies that make translating these results and determining an overall conclusion on the effects of radiation on articular cartilage.

A major inconsistency in the literature is the type of animal/cell model used in each study. There were at least seven different models found in current literature and these models can be broken up into macroscopic and microscopic models. The majority of the models were microscopic. These models focused on the articular chondrocytes found in cartilage, but where these articular chondrocytes were harvested from was from four different sources: primary cultured adult human articular cartilage cells, primary cultured rat articular cartilage cells, and primary rat costochondral cells [13, 15, 26, 37, 44]. In addition, most were cultured on 2D substrates which are known to cause chondrocytes to dedifferentiate. Another microscopic model used was human chondrosarcoma cells. There were similar issues with the variety of the macroscopic models used in studies. We noted three types of macroscopic models commonly found: a porcine model, an adolescent male rat model, and a rabbit model. Overall, since there were a wide variety of models used, it was challenging to take results from these studies, compare them, and make a general overall conclusion about the effects of radiation on articular tissue and cells.

Not only were there variability on the models used, there was also large variance in the type of radiation and the doses used. However, the amount of studies examining the effects of each type of ionizing radiation has not been consistent. The majority of studies examine the effects of X-ray radiation and a handful of studies examine gamma radiation effects, whereas very few discuss the effects of particulate radiation. Some studies did not report the type of radiation used and those studies that did report the type of radiation, did not always report the radiation source. It is important to know the type of irradiator used since the source directly affects the radiation energy and dose rate delivered as well as the uniformity of the dose [49].

In order to compare results between studies, it is also imperative to know the dose rate that the experiment used. However, the majority of studies did not report the dose rate used. There is a direct relationship between the dose rate and the fraction of cells that are killed by the given dose in the dose ranges of radiotherapy [50]. This is due mainly to the ability of the cells to repair the sub-lethal damage that occurs during radiotherapy [50]. Therefore, reporting the dose rate aids in making the results more translatable to other studies as well as to making overall conclusions.

The final major difference among the studies was the total dose used. Older studies focused on high-doses of radiation, doses greater than 3Gy, due to the release of the atomic bomb in 1945 [15]. However, more modern studies that suggest the study is focusing on low-dose radiation may actually be using doses that are considered moderate to high-doses of radiation. This may be due to medical procedures and fractionated radiotherapy regimens using fractions of 3Gy, since 3Gy doses are considered to be more manageable doses for the body to heal from [50]. Therefore, in order to determine the effects of low-dose radiation on articular cartilage, it is essential that the definition of low-dose be universally accepted. Generally, doses lower than 2Gy are considered low-doses of radiation. However, some studies state that 3Gy is a low-dose and others state that it is a high-dose.

However, despite the variability in the types of models, the types of radiation, the dose rates, and the doses used, there is one consistent conclusion that the majority of studies have stated. The majority of modern studies have concluded that more research must be conducted in order to determine the effects of low-dose radiation on articular cartilage. Therefore, there are still inconsistencies and a significant gap in literature in regard to low-dose radiation and its effects on articular cartilage.

## Abbreviations

Bq: Becquerel
Gy: Gray
min: minute
Sv: Sievert
